# Juvenile polyposis syndrome with gastric and duodenal polyposis presenting with refractory anemia and protein-leakage gastroenteropathy in a patient with *SMAD4* mutation: a case report

**DOI:** 10.1186/s40792-023-01796-4

**Published:** 2024-01-09

**Authors:** Kenya Nakamura, Koji Kubota, Akira Shimizu, Tsuyoshi Notake, Tomohiko Ikehara, Kentaro Umemura, Atsushi Kamachi, Takamune Goto, Hidenori Tomida, Yoshiyuki Takahashi, Tadanobu Nagaya, Takeji Umemura, Yuji Soejima

**Affiliations:** 1 Division of Gastroenterological, Hepato-Biliary-Pancreatic, Transplantation and Pediatric Surgery, Department of Surgery, Shinshu University School of Medicine, 3-1-1 Asahi, Matsumoto, Nagano 390-8621 Japan; 2 Department of Gastroenterology, Shinshu University School of Medicine, Matsumoto, Japan

**Keywords:** Juvenile polyposis syndrome, Pancreaticoduodenectomy, Total gastrectomy, Refractory anemia, Protein-losing gastroenteropathy

## Abstract

**Background:**

Juvenile polyposis syndrome (JPS) is an autosomal dominant, inherited disorder characterized by multiple hyperproliferative polyps of the gastrointestinal tract, particularly of the colon, rectum, and stomach. *SMAD4* mutations are frequently associated with multiple polyposis of the stomach; the condition causes severe bleeding and hypoproteinemia, which may progress to severe dysplasia and adenocarcinoma formation. We report our experience with the first case of total gastrectomy with pancreaticoduodenectomy following two partial jejunectomies for JPS, who presented with refractory anemia and protein-losing gastroenteropathy due to polyposis of the stomach and duodenum.

**Case presentation:**

A 33-year-old Japanese man presented with the chief complaint of shortness of breath on exertion. His family history included gastric polyposis (mother, aunt, and cousin) and cerebral infarction (grandmother). Blood testing at the initial visit indicated iron-deficiency anemia, whereas endoscopy revealed multiple polyps in the duodenum and jejunum. Genetic testing revealed a 4 bp deletion (TGAA) in exon 5 of the *SMAD4* gene; two partial small bowel resections were performed, but polyps grew in the remaining stomach, duodenum, and small intestine. The patient developed hypoalbuminemia and anemia, and required central venous nutrition and blood transfusion. However, because the hyponutrition and anemia remained poorly controlled, a total gastrectomy with concomitant pancreaticoduodenectomy was performed. Malnutrition and anemia improved, and there was no polyp recurrence in the remaining intestinal tract at 18 months after the surgery.

**Conclusions:**

We report a case of JPS with refractory anemia and protein-losing gastroenteropathy that was treated with total gastrectomy with concomitant pancreaticoduodenectomy. Although the surgery was highly invasive, the patient’s nutritional status and anemia improved postoperatively, and the treatment was successful. However, to determine the appropriate surgical procedure, a detailed examination of the gastrointestinal lesions and the effects of the surgical invasion on nutritional status must be undertaken.

## Background

Juvenile polyposis syndrome (JPS) is an autosomal dominant, inherited disorder characterized by multiple hamartomatous polyps in the gastrointestinal tract, especially in the stomach, small intestine, colon, and rectum [1, 2]. Approximately 40%–60% of the patients with JPS have germline mutations in the *SMAD4* or *BMPR1A* genes [1, 2]; non-familial cases have also been reported [3]. Though JPS polyps are often benign, they could turn malignant [1, 2, 4]. *SMAD4* mutations are often associated with multiple polyposis of the stomach, which causes severe bleeding and hypoproteinemia; severe dysplasia and adenocarcinoma are observed in rare cases [4, 6]. We report the first case of total gastrectomy (TG) and pancreaticoduodenectomy (PD) performed (following two partial jejunostomy procedures) in a patient with JPS, refractory anemia, and protein-losing gastroenteropathy caused by polyposis of the stomach and duodenum. We present the postoperative course of the patient, focusing on their nutritional status.

## Case presentation

**Fig. 1:**
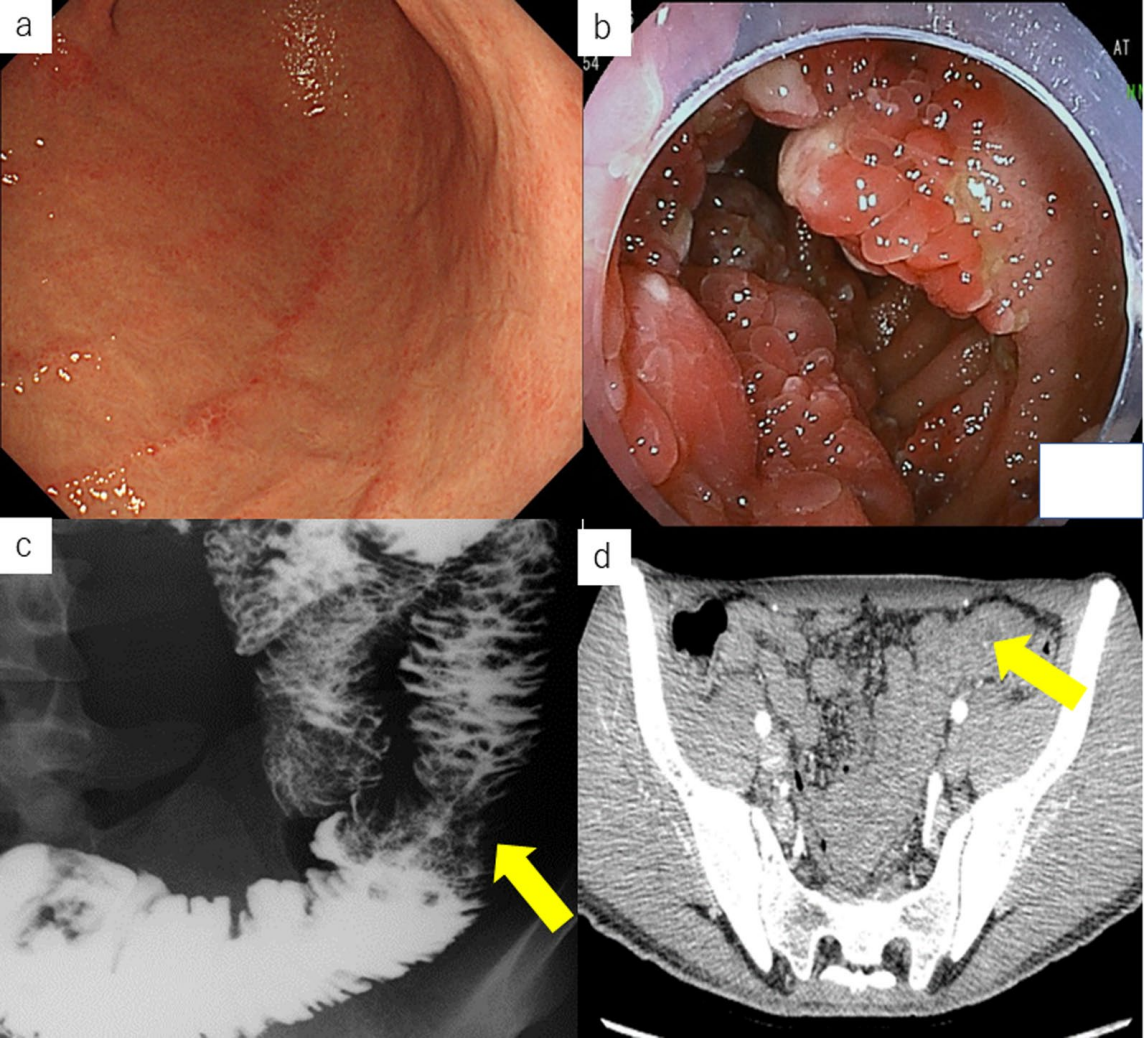
Imaging findings before the first surgery. **a** Esophagogastroduodenoscopy reveals no lesions in the stomach. **b** Capsule endoscopy reveals multiple polyps in the jejunum. **c** Small bowel series reveal multiple translucencies in the jejunum (arrow). **d** Abdominal contrast-enhanced computed tomography reveals diffuse wall thickening of the jejunum (arrow)

A 33-year-old Japanese man presented to the department of gastroenterology in our institution with the chief complaint of shortness of breath on exertion. His past medical history was unremarkable; however, he had a family history of polyposis of the stomach (mother, aunt, and cousin) and cerebral infarction (grandmother). He had a history of occasional drinking and smoking (4–5 cigarettes/day for 6 months). Blood tests at the initial visit revealed iron deficiency anemia. Esophagogastroduodenoscopy (EGD), colonoscopy (CS), and capsule endoscopy revealed multiple polyps in the region extending from the duodenum to jejunum (Fig. 1). Biopsy revealed cystic dilation of the glandular ducts with inflammatory cell infiltration. Furthermore, genetic testing revealed a 4 bp deletion (TGAA) in exon 5 of the *SMAD4* gene.

**Fig. 2:**
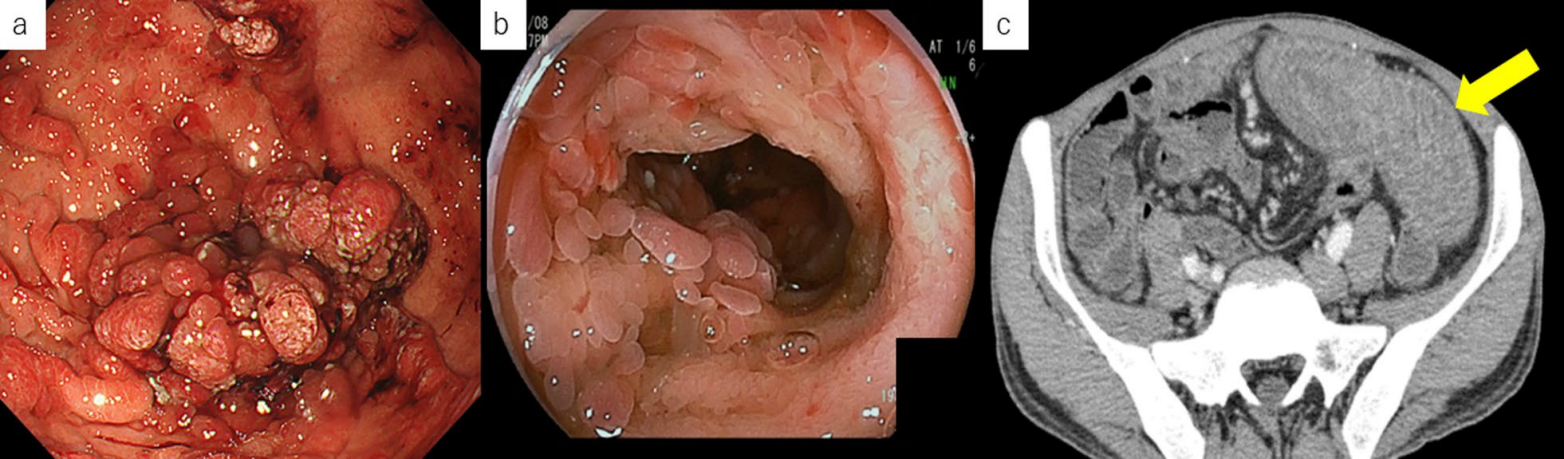
Imaging findings before the second surgery. **a** Esophagogastroduodenoscopy reveals multiple polyposis in the stomach. **b** Capsule endoscopy reveals recurrent polyposis in the jejunum. **c** Abdominal contrast-enhanced computed tomography reveals recurrence of diffuse wall thickening in the jejunum (arrow)

**Fig. 3:**
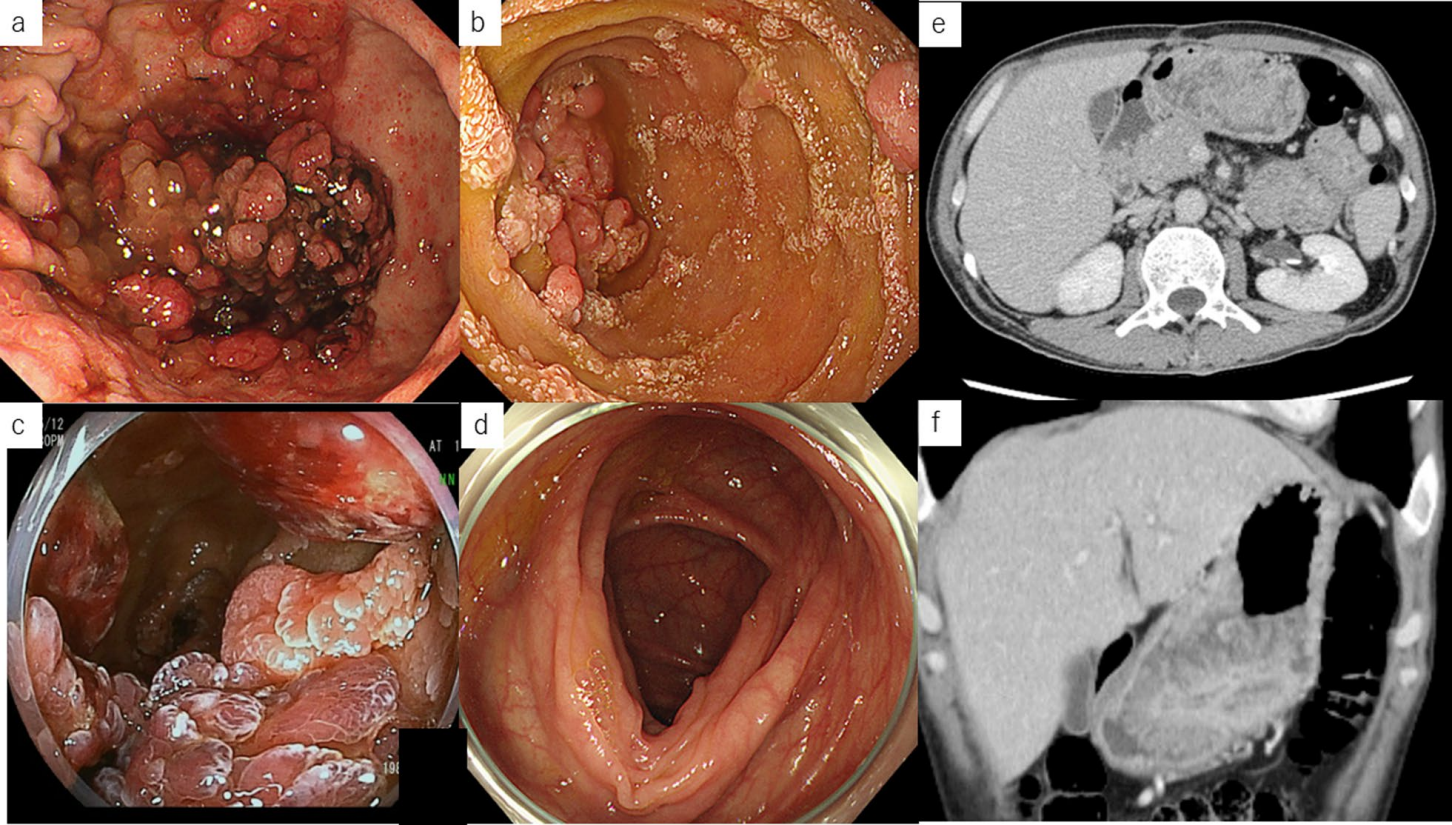
Imaging findings before total gastrectomy and pancreaticoduodenectomy. **a** Esophagogastroduodenoscopy reveals increased polyposis in the stomach as well as hemorrhage. **b** Esophagogastroduodenoscopy reveals polyposis in the duodenum. **c** Capsule endoscopy reveals polyposis extending from the third portion of the duodenum to the jejunum. **d** Colonoscopy reveals no polyps in the colon. **e**, **f** Abdominal contrast-enhanced CT scan reveals wall thickening with contrast from the stomach and third portion of the duodenum to the jejunum

**Fig. 4:**
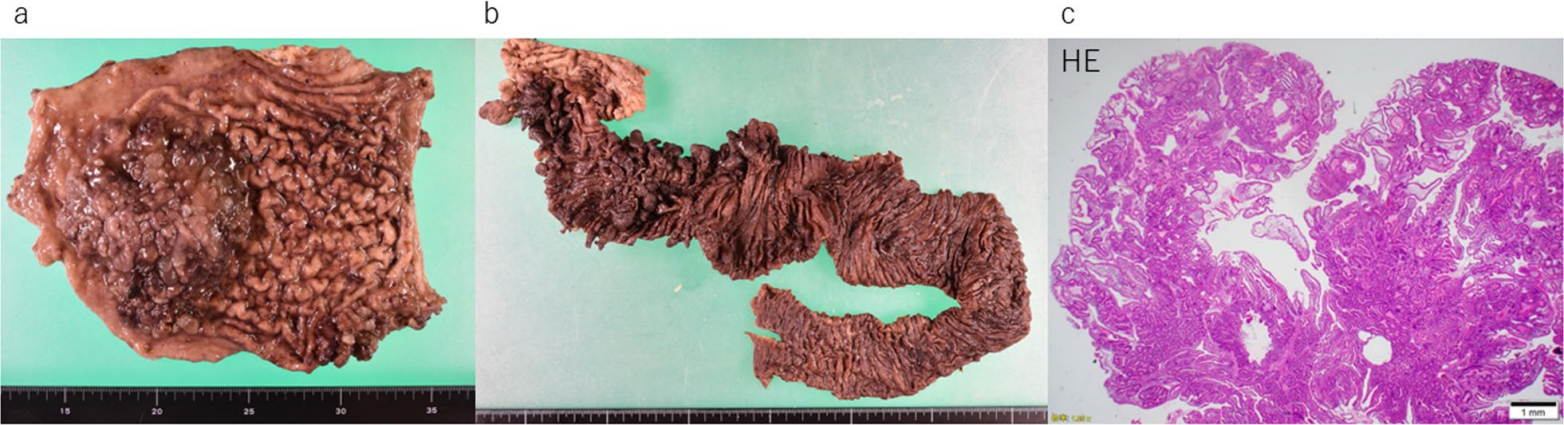
Pathological findings. **a**, **b** Macroscopic findings. **c** Microscopic findings. **a** Multiple polyps are seen in the antrum. **b** Multiple polyposis is observed, extending from the duodenum to the jejunum. **c** Hamartomatous polyps are observed with inflammatory cell infiltration and dilation of the glandular ducts. No malignant findings are noted

**Fig. 5:**
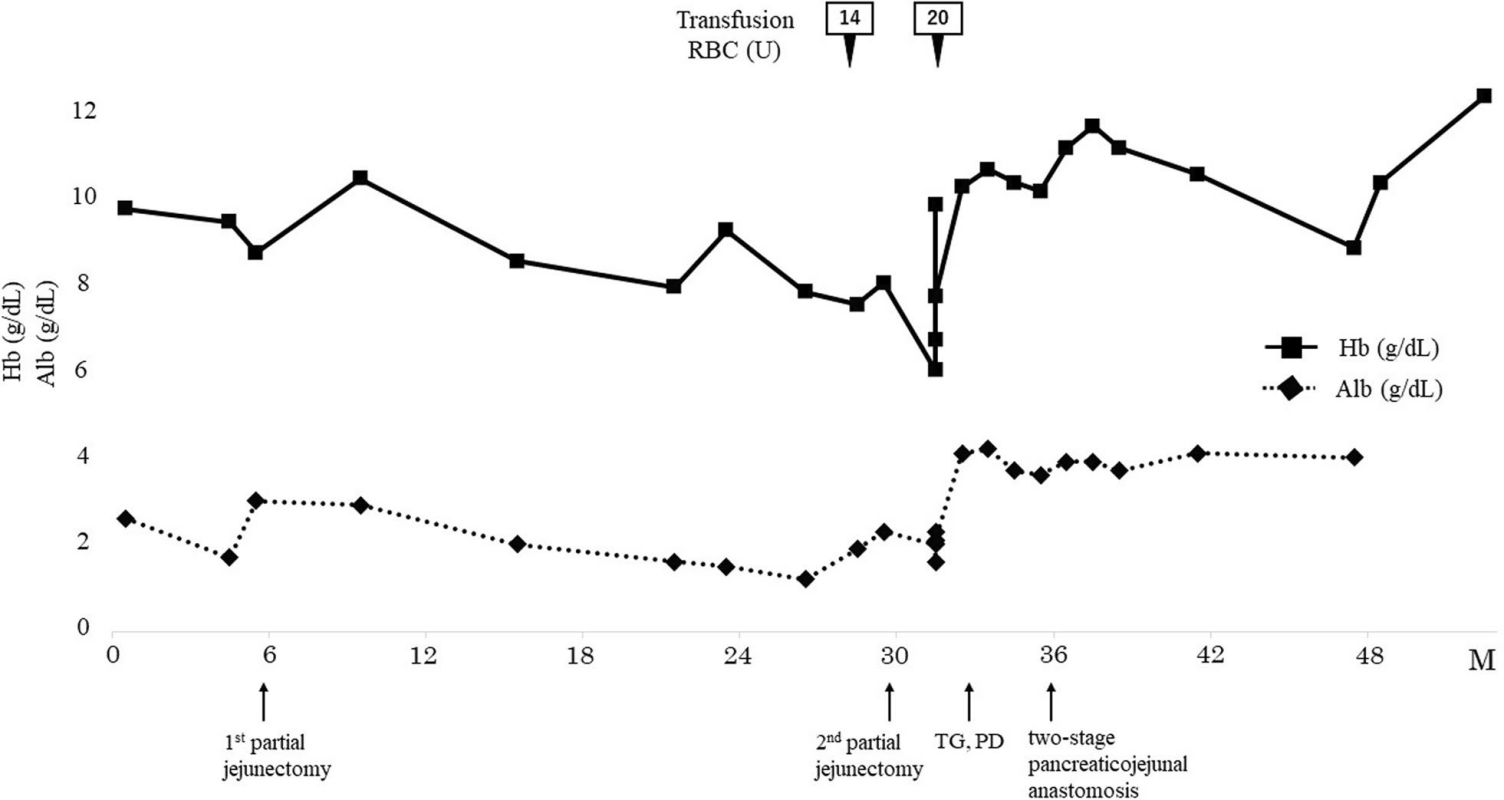
Clinical course of the treatment and changes in the serum hemoglobin and albumin levels. *Hb* hemoglobin, *Alb* albumin, *TG* total gastrectomy, *PD* pancreaticoduodenectomy, *RBC* red blood cell

Based on the aforementioned findings, the patient was diagnosed as having protein-losing gastroenteropathy associated with inflammation from the duodenum to the jejunum due to JPS. Because medical therapy was ineffective, approximately 25 cm of the most inflamed portion of the jejunum was partially resected. His symptoms improved temporarily; however, the inflammation of the intestinal tract worsened again, and the albumin level dropped to 1 mg/dL due to protein-losing gastroenteropathy. A totally implantable central venous access port was placed, and central venous nutrition was started; however, the albumin level did not improve, and persistent pain secondary to small intestinal inflammation reappeared. The polyps in the stomach and duodenum, and especially those in the jejunum, were enlarged (Fig. 2); hence the symptoms were considered to have been caused by the polyps in the jejunum. Accordingly, 2 years and 2 months after the initial surgery, a partial jejunectomy of approximately 72 cm was performed. Unfortunately, the surgery was ineffective. His height, weight, and body mass index were 182 cm, 56 kg, and 16.9 kg/m^2^, respectively; these were indicative of emaciation. Three years earlier, his weight was 77 kg, indicating a weight loss of 21 kg in 3 years. Marked iron-deficiency anemia (hemoglobin, 6.0 g/dL; Fe, 27 μg/dL; ferritin, 13.0 ng/mL; total iron binding capacity 242 μg/dL; and unsaturated iron binding capacity, 215 μg/dL) and hypoproteinemia (albumin, 2.0 g/dL) associated with protein-losing syndrome were also observed. Thus, frequent blood transfusions were required to alleviate the symptoms. No abnormalities were noted in the tumor marker levels; furthermore, EGD performed 31 months after the first EGD revealed multiple rapidly enlarging polyps, bleeding in the stomach, and a black residue in the stomach indicative of chronic bleeding (Fig. 3a, b). Double-balloon enteroscopy revealed a cluster of polyps in the jejunum, at 15 cm anal side from the ligament of Treitz; these were only scattered on the anorectal side of the same area (Fig. 3c). CS revealed a single polyp and no apparent abnormality in the mucosa (Fig. 3d). Abdominal computed tomography (CT) showed wall thickening in the stomach and from third portion of the duodenum to the jejunum (Fig. 3e, f). No ascites was observed. There were no findings suggestive of hereditary hemorrhagic telangiectasia, such as pulmonary or hepatic arteriovenous fistulas. Tc-99 m-human serum albumin dextran protein leak scintigraphy revealed an accumulation in the small intestine at 2 h and 4 h on planar images, while single-photon emission CT revealed an accumulation in the small intestine, indicating protein-losing gastroenteropathy. Therefore, TG and PD were performed for JPS caused by the *SMAD4* mutation, which presented with anemia and protein-leakage gastroenteropathy that were difficult to treat medically. Because the patient was undernourished and considered as having high risk of pancreatic fistula formation, complete external drainage of pancreatic juice with the intention of subsequently performing second stage pancreatojejunostomy was applied during the first stage surgery. Surgical findings indicated an anastomosis in the jejunum located 40 cm from the Treitz ligament, and the jejunal wall was thickened to that point. The stomach was dilated, and polyps were palpated in clusters throughout the stomach, especially in the lower part of the gastric body. The jejunum was resected approximately 60 cm anorectally from the Treitz ligament, and the remnant small intestine measured approximately 320 cm. The reconstruction methods used were variants of Roux-en-Y reconstruction and Child’s reconstruction. Gross examination revealed multiple clusters of polyps in the stomach and duodenum (Fig. 4a, b) and pathological findings showed prominent glandular epithelium forming an elevated lesion with hyperplastic changes (Fig. 4c). The glandular ducts were dilated and irregularly branched, and the mucosal lamina propria was edematous (Fig. 4c). These findings were indicative of JPS; however, no malignancy was observed. The postoperative course was uneventful, and the patient was discharged on postoperative day 47. At four months after the first stage surgery, a second stage pancreatojejunostomy was performed. At 1 year and 6 months postoperatively, there was no evidence of worsening polyps, anemia, or nutritional status (Fig. 5). Because this disease is hereditary, his older sister underwent complete examination and was diagnosed with JPS. She has been prescribed regular check-ups with a gastroenterologist.

## Discussion

With an incidence of 1 in 100,000–160,000 people, JPS is a rare disease in which multiple malignant polyps occur throughout the gastrointestinal tract [2, 4, 6]. It is a genetic disorder with an autosomal dominant form of inheritance, and pathological variants in the *SMAD4* gene (17%–35%) or the *BMPR1A* gene (17%–25%) have been established as the cause [1, 2, 4–8]. Both mutations account for approximately 60% of the genetic variants in JPS; approximately 25% are isolated cases with no family history [1–3]. As observed in this patient, pathological *SMAD4* mutations are characterized by polyposis of the stomach, which may be accompanied by lesions of the upper gastrointestinal tract. In JPS, the polyps are found in all regions of the gastrointestinal tract: colon (98%), stomach (14%), small intestine (7%), and duodenum (2%; with overlap) [4, 6, 9]. The average age of symptom onset is reported to be 22 years [4]. Therefore, it is recommended that patients with a family history of JPS undergo upper and lower endoscopy beginning at age 12–15 years [6]. In this case, an unnoticed polyp in the stomach appeared and enlarged 31 months later. As polyps are found in young people, frequent examination may be difficult, but it is impossible to predict when and where polyps will appear. Regular endoscopic examinations are necessary every two to three years, even in areas thought to be free of polyps. According to the site of origin, JPS is classified into generalized juvenile polyposis (GJP), juvenile polyposis coli (JPC), and juvenile polyposis of the stomach (JPST) [6]. JPST is more common in Asia due to the high prevalence of *SMAD4* variants [6, 10]; in Japanese studies, JPST accounted for 36.3% of patients with JPS, comparable to 36.3% with JPC and less than 27.4% with GJP, while *SMAD4* variants were found in 75% of the patients tested for the mutation [6]. In contrast, JPC was the most common in the UK study at 57%, followed by GJP at 36%. Despite the most common genetic variant being SMAD4, the site of polyp expression varied by race [4]. Because the patient in the present case presented with polyposis distributed in the duodenum and jejunum in addition to the gastric lesions, we classified the condition as GJP instead of JPST. These findings make this a rare and valuable case.

The mutation is commonly present in exon 5 of the *SMAD4* gene. However, even if the clinical symptoms and site of polyp development differ among cases with the same gene mutation site; thus, it is necessary to consider the follow-up and treatment methods independently. Our patient’s older sister has the same disease with an almost identical distribution of polyps; this makes these findings remarkable.

Endoscopic resection is the first choice of treatment for hamartomatous polyps because they are benign. Surgical resection is indicated in cases with larger and more polyps, invasive cancer, intestinal accumulation, poorly controlled anemia, and hypoproteinemia. The incidence of cancer in patients with JPS is 11%–86%, with a significantly higher incidence of gastric cancer in those with *SMAD4* mutations (10.3%); preventive gastrectomy is recommended in such cases [4, 6, 9, 11]. A PubMed search using the keywords “juvenile polyposis syndrome” and “pancreaticoduodenectomy” revealed no reports. Thus, to the best of our knowledge, this is the first case in which PD was performed for JPS. In this case, although polyps in the stomach and duodenum were identified in the middle of the process, TG and PD were considered highly invasive procedures. These procedures cause nutrient malabsorption and have the potential to induce hyponutrition and anemia. The decision to perform a minimally invasive partial resection of the small intestine as the initial surgical procedure was appropriate, because no cases of PD have been reported. However, minimally invasive surgeries (such as partial jejunectomy) and medical treatments (such as central venous nutrition) could not sustain daily life and significantly compromised the patient’s quality of life. There were no suitable treatment alternatives other than a highly invasive surgery. Based on previous operative, endoscopic and imaging findings, we were confident that the ileum would be adequately preserved. In addition, as the pre-albumin levels were normal, absorption and synthesis capacity were also expected to be normal. Therefore, TG and PD were performed in the hope of improving nutritional status. However, invasive surgery with simultaneous PD should be carefully assessed for indications and may lead to adverse outcomes. The patient's nutritional status and anemia should continue to be monitored regularly. In addition, CS should be performed once every 1–3 years due to the possible appearance of lesions in the colon.

## Conclusions

We report a case of JPS with refractory anemia and protein-losing gastroenteropathy that was treated with TG and PD. Although the surgery was highly invasive, the patient's nutritional status and anemia improved postoperatively, and the treatment was successful. However, a thorough examination of gastrointestinal lesions and symptoms is necessary to determine the appropriate surgical procedure.

## Data Availability

All data generated during case management are included in the published article.

## Abbreviations

JPS: Juvenile polyposis syndrome
TG: Total gastrectomy
PD: Pancreaticoduodenectomy
EGD: Esophagogastroduodenoscopy
CS: Colonoscopy
CT: Computed tomography
